# ClickDiary: Online Tracking of Health Behaviors and Mood

**DOI:** 10.2196/jmir.4315

**Published:** 2015-06-15

**Authors:** Ta-Chien Chan, Tso-Jung Yen, Yang-Chih Fu, Jing-Shiang Hwang

**Affiliations:** ^1^ Research Center for Humanities and Social Sciences Academia Sinica Taipei City Taiwan; ^2^ Institute of Statistical Science Academia Sinica Taipei City Taiwan; ^3^ Institute of Sociology Academia Sinica Taipei City Taiwan

**Keywords:** health behaviors, mood, diet, physical exercise, quality of sleep, personality

## Abstract

**Background:**

Traditional studies of health behaviors are typically conducted using one-shot, cross-sectional surveys. Thus, participants’ recall bias may undermine the reliability and validity of the data. To capture mood changes and health behaviors in everyday life, we designed an online survey platform, ClickDiary, which helped collect more complete information for comprehensive data analyses.

**Objective:**

We aim to understand whether daily mood changes are related to one’s personal characteristics, demographic factors, and daily health behaviors.

**Methods:**

The ClickDiary program uses a Web-based platform to collect data on participants’ health behaviors and their social-contact networks. The name ClickDiary comes from the platform’s interface, which is designed to allow the users to respond to most of the survey questions simply by clicking on the options provided. Participants were recruited from the general population and came from various backgrounds. To keep the participants motivated and interested, the ClickDiary program included a random drawing for rewards. We used descriptive statistics and the multilevel proportional-odds mixed model for our analysis.

**Results:**

We selected 130 participants who had completed at least 30 days of ClickDiary entries from May 1 to October 31, 2014 as our sample for the study. According to the results of the multilevel proportional-odds mixed model, a person tended to be in a better mood on a given day if he or she ate more fruits and vegetables, took in more sugary drinks, ate more fried foods, showed no cold symptoms, slept better, exercised longer, and traveled farther away from home. In addition, participants were generally in a better mood during the weekend than on weekdays.

**Conclusions:**

Sleeping well, eating more fruits and vegetables, and exercising longer each day all appear to put one in a better mood. With the online ClickDiary survey, which reduces the recall biases that are common in traditional one-shot surveys, we were able to collect and analyze the daily variations of each subject’s health behaviors and mood status.

## Introduction

Happiness has been regarded as an important indicator correlated to an individual’s mental and physical health [1,2]. Although it is well known that emotional state is an important piece of information to consider in health or psychological studies, such information has been either overlooked [3] or confounded by one’s daily activities [4], social contacts [5], personal health behaviors [6,7], and personality [8]. In conventional surveys, for example, respondents are often asked how happy or unhappy they have been, in general, over a long period of time, such as the past month or the past year [9], a time frame that is often too vague or too long to recall specific details. The information collected from such surveys can be biased by more recent and memorable experiences, or confused with the participant’s general mood, both undermining the extent of accuracy in data analysis. In some cases it may also be difficult for researchers to determine which factors play more critical roles in distinguishing one’s mood. One way to overcome such limitations is to get each participant’s dynamic daily mood changes properly documented.

In an effort to collect such longitudinal data about mood changes, some health studies use a journal-like design, on a daily basis, to tap participants’ emotional status [10], headache symptoms (of children) [11], signs of depression and stress from working or learning [12], and instances of gastrointestinal illness and other physical illnesses [13]. Data collection in such studies usually lasts for only 1 week or 1 month, thus limiting their sample sizes despite having repeated measurements. Although some social network studies also use the contact diary format to collect data [14-16], they all rely on conventional paper-and-pencil instruments. To improve the process of diary taking, minimize participants’ efforts, and enhance the accuracy of results, we designed an online-based diary platform for our study.

In previous studies, one’s emotional stability has been linked to certain personal characteristics, such as personality—particularly agreeableness and neuroticism [8]—and demographic factors, such as age [17]. Those individual factors, however, are more or less fixed, either ascribed or predetermined since childhood. We believe that it is also important to examine how emotional stability or happiness varies by other variables that are more closely relevant to one’s health behaviors or lifestyles, such as physical exercise [2,18], quality of sleep [19], consumption of vegetables and fruits [6,7], and so on.

The aim of this study is to use the online diary platform to collect health and contact data on a daily basis. We want to elucidate the extent to which a person’s daily mood varies by his or her personal characteristics, demographic factors, and health behaviors, as well as the day of the week and the extent of social interactions in everyday life.

## Methods

### Ethics

This study was approved by the Institutional Review Board (IRB) on Humanities and Social Science Research, Academia Sinica (AS-IRB-HS 02-13022). Participants must be at least 20 years old and capable of making juridical acts in Taiwan. Before registering as a participant of the ClickDiary program, one must read the guidelines of the program and give informed consent. We have removed personal identifiers, such as names and email addresses, and assigned serial numbers to both the participants and everyone in their contact networks to ensure privacy.

### The ClickDiary Program

**Figure 1:**
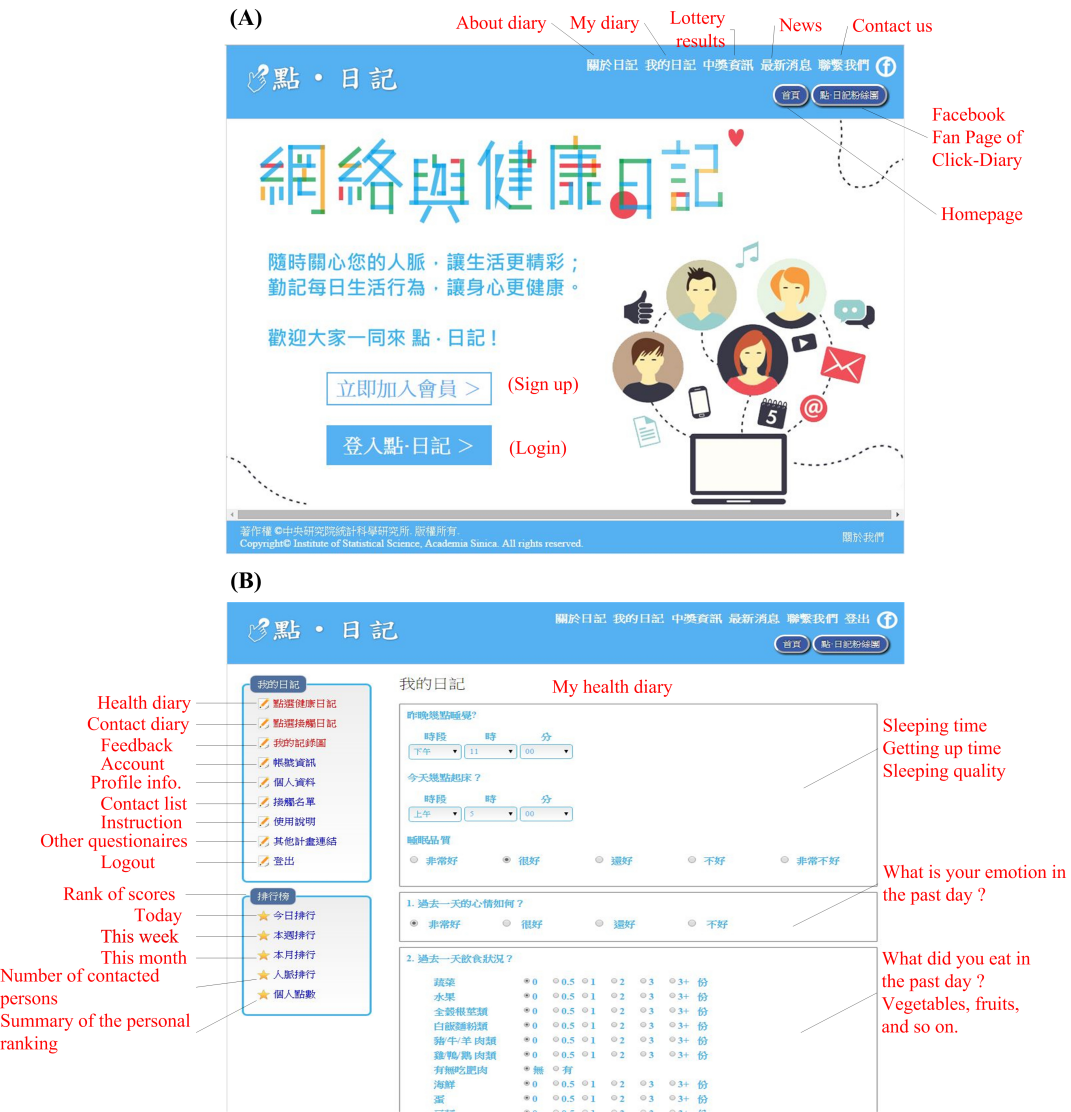
Screenshots of ClickDiary. (A) Home page; (B) The interface of the health diary.

The ClickDiary program uses a Web-based platform [20] (see Figure 1, section A) to collect data on participants’ health behaviors and their social-contact networks. The interface of ClickDiary is designed so that users can input their responses by clicking options in the instrument, making it easier to record responses on a daily basis (see Figure 1, section B). At this stage, this program is specifically tailored to the Taiwanese population. The program is unlike traditional cross-sectional health behavior surveys [21] or one-shot paper-and-pencil contact surveys [15,16]. In addition to being user friendly, the ClickDiary program helps generate daily, real-time, longitudinal data. After participants successfully sign up for their accounts, they are asked to provide demographic information, including age, gender, place of residence, marital status, and current job. The program also collects the Big Five personality traits—openness, conscientiousness, extraversion, agreeableness, neuroticism (OCEAN) [14,22], whose exact wording was taken from the International Personality Item Pool (IPIP) [23]—height and weight for calculating body mass index (BMI, kg/m^2^), perceived health status and happiness, the number (and characteristics) of people contacted during the day, and a baseline health survey, which borrows items from the Taiwan Social Change Survey [24]. After giving such basic information, participants can then proceed to fill out their health diary and contact diary. A reward system serves as incentive for the participants to keep both diaries at least three times a week. In the following sections we introduce our health diary, recruitment methods, quality-control process, reward system, and feedback design.

### Health Diary

In this study, we used only the health diary and baseline profile data for our analysis. Thus, we will not introduce the contents of the contact diary here. In the health diary, we asked participants seven major questions regarding what happened in the past day, including their sleep behaviors (ie, what time they got up and went to bed, and how well they slept—very good, good, fair, poor, or very poor), their mood during the past day (very good, good, fair, or poor), their food intake during the past day, duration of physical exercise (no exercise, 1 to 30 minutes, 31 to 60 minutes, 61 to 120 minutes, or longer than 120 minutes), the number of people with whom they had contact in the past day, the number of suspected or confirmed influenza-inflicted people (and their symptoms) with whom they had contact, and the maximum distance they traveled from their residence (less than one kilometer, 1 to 9 kilometers, 10 to 49 kilometers, 50 to 300 kilometers, or farther than 300 kilometers).

In the section on food intake, we listed 16 categories of food, the quantities of which were measured in six degrees (ie, 0, 0.5, 1, 2, 3, and 3+) with different units. The 16 categories of food were vegetables, fruits, whole grains and rootstock, rice and flour, pork/beef/mutton, chicken/duck/goose, fatty meat, seafood, eggs, beans, milk and cheese, nuts, fried foods, processed foods, desserts, and sugary drinks. For this study, we selected and consolidated the food types into the following eight types: meat, seafood, milk and cheese, whole grains and rootstock, fried foods, sugary drinks, desserts, and vegetables and fruits. Mouse-over labels were available to inform users about the units for the different categories of food. One serving of fruit, for example, was equal to a fistful or 80% of a 240 milliliter bowl. These hints ensured that all participants had the same understanding of the units.

### Time Definition

We designed the health diary to collect data from the past 24 hours. Because it was likely that participants would enter data several times a day, we reorganized the dates indicated on the diaries according to the logged time. If the logged time fell after noon of the current day, the entry would be included in the current day’s health diary. If the logged time fell between noon of the previous day and noon of the current day, then it was considered the previous day’s health diary entry.

### Recruitment of Participants

Recruited from various channels, our participants included university students, school teachers and administrative employees, volunteers at health-promotion centers, hospital patients, and community college students, as well as other adults in the general population. Due to a limited budget and the longitudinal nature of the ClickDiary program, we were unable to recruit a representative sample as large as that of the one-shot cross-sectional surveys. To diversify the patterns of participation, however, we used two major recruitment strategies. First, we targeted unspecified individuals and groups to solicit volunteers using posters and other promotional campaigns, both online and offline, such as on Facebook and in classes held at different universities and community colleges scattered across northern, central, and southern Taiwan. Second, in several groups with delineated boundaries, we asked group leaders to monitor the participation rate by periodically encouraging group members to complete both health and contact diaries. To help such monitoring, our system issued a summary report of each group to its leader at the end of each week. If a group’s diary entries met our standards for both quantity and quality, we sent out convenience store vouchers to the leader and each group member as a reward.

### Quality Control

To ensure the quality of the data, we checked the data pattern of each participant every week. If we suspected that a specific participant had not been keeping his or her diaries properly, the participant’s serial number was put on an alert list, and any data entered by the participant would be excluded from our analysis. In addition, anyone who provided incomplete and poor data was excluded from the random drawing and from receiving any reward. We did not publish details of our quality-control procedure, because quality control might become more difficult if the participants get acquainted with our rules of screening.

### Incentives

We designed a random drawing that took place both weekly and monthly. Each entry into the drawing was assigned a weight based on each participant’s corresponding weekly and monthly cumulative points. The more time and effort participants spent on completing their ClickDiary entries, the greater were their chances to win a reward. A participant automatically received 20 points after finishing the health diary each day. If someone submitted multiple health diary entries within a single day, the system still gave the participant only 20 points. Participants also received 3 points for each contacted person entered into the contact diary. Although one could record multiple interactions with the same contacted person on the same day, each contacted person would only yield 3 points. To prevent the participants from intentionally giving false data about daily contacts, we developed a screening program for checking the accuracy and quality of diary data every week.

### Feedback

We did not give feedback to participants, based on the IRB’s recommendations. We did, however, provide an interactive Web chart summarizing the records in each participant’s contact and health diaries. Participants could then view the summarized reports that kept track of their health behaviors for up to one year. In addition, participants could gain insight from their overall contact patterns by checking the contact network tree we developed, which also allowed them to take a glance at how their mood changed when contacting different people over time.

### Statistical Analysis

Participants’ reports on their overall mood in the past 24 hours were coded on a 4-point scale—1 (Poor), 2 (Fair), 3 (Good), and 4 (Very good). Other health-related daily entries included diet, exercise, and the time and quality of sleep during the 184-day study period. For this study, we applied a multilevel proportional-odds mixed model to analyze the data [25,26]. With this model, we analyzed the relationship between mood swings and health behaviors, while adjusting for individual characteristics, such as age, gender, and personality.

Let Y_ij_ be the i^th^ individual’s scalar response of mood recorded on day t_ij_, where j=1,2,...,T_i_, and T_i_ ranged from 26 to 184 for the n=130 participants. The random-effects ordinal regression model for analyzing such multilevel data is given as:

logit[Pr(Y_ij_≤k)]=θ_k_ – a_i_ – b_1_∙I (Y_i,j-1_=1) – b_2_∙I (Y_i,j-1_=2) – b_3_∙I (Y_i,j-1_=3) – b_4_∙I (Y_i,j-1_=4) –α’Z_i_ – β’X_ij_, k=1,2,3

Time-dependent covariates for the i^th^ participant are denoted by X_ij_ = (X_1ij_,...,X_qij_)’ and the other covariates are denoted by Z_i_ = (Z_1i_,...,Z_pi_)’. The parameters {θ_k_} increasing in k are known as thresholds or cut-points. Random components of a_i_ ~ N(0,σ^2^_a_), representing variations of these logits for each response level k among the n individuals, are added into the mean equation to adjust for the thresholds of each individual.

For the repeated recordings, each individual’s scalar response may be associated with previously reported responses. To take this into account, we added another random component b_l_ ~ N(0, σ^2^_b_), l=1,...,4 to the model for further adjustment of the thresholds. This model has the same fixed effects as determined by the parameters α and β of the covariates of interest for each response level k. For example, the ratio between the odds of making a response at each level k or below with covariates X_ij_ = x^1^_ij_ and x^0^_ij_ is exp[–β’(x^1^_ij_ - x^0^_ij_)]. In this application, our covariates are all categorical variables. For the h^th^ covariate of interest, X_hij_, which is represented by an indicator variable corresponding to a level of a categorical variable, such as the quality of sleep, we report exp(–β_h_) as odds ratio (OR) of cumulative probabilities between the presence of the level and the baseline level of this categorical variable.

We might expect that an individual with the level of this categorical variable had odds of worsening mood exp(–β_h_) times compared to those with a baseline level of this variable if the odds ratio were larger than 1. On the contrary, an estimate of exp(–β_h_)<1 indicates better mood with the level of this categorical variable. We used the clmm function from the R package “ordinal” to estimate the model parameters [27].

## Results

We selected 130 participants from 726 qualified participants (17.9%) who had completed at least 30 days of contact diaries from May 1 to October 31, 2014. The contact diary served better as our criterion because it required more time to complete—on average, it took 1 minute and 12 seconds to record all variables per contact, or about 12 minutes for 10 contacts per day—than the health diary, which averaged 1 minute and 39 seconds per day. Thus, a more complete contact diary normally indicates higher commitment by the participant, which in turn ensures that the health diary, as well as other variables, are of better quality.

The average participant kept the health diary for about 69 days, with a range from 26 to 184 days (see Table 1). The 130 participants had input 8824 complete health diary entries. Our sample was overrepresented by females (98/130, 75.4%), and the overall mean age was 33.1 (SD 13.4), ranging from 20 to 67 years. Participants averaged a BMI of 22.0 kg/m^2^ (SD 3.3), and most were within the normal range—healthy BMI: 18.5 kg/m^2^≤BMI<24 kg/m^2^. Among the Big Five personality items, about 90% of participants said they were thorough in performing a task—a measure of being conscientious—or they sympathized with others’ feelings—a measure of being agreeable (see Table 2). We used the 10 personality measures as covariates for the multilevel proportional-odds mixed model.

**Table 1:** Summary of selected variables for the 32 male and 98 female participants (n=130).

Variable	Mean (SD)	Minimum	Maximum
Participating days	68.9 (38.1)	26	184
Age in years	33.1 (13.4)	20	67
Body mass index (kg/m^2^)	22.0 (3.3)	16.2	36.0

**Table 2:** Summary of personality items for the 32 male and 98 female participants (n=130).

Big Five personality items		Responses			
		Not at all, n (%)	Not very, n (%)	Somewhat, n (%)	Very, n (%)
**Extraversion**					
	Outgoing and sociable	13 (10.0)	47 (36.2)	58 (44.6)	12 (9.2)
	Do not talk a lot	22 (16.9)	56 (43.1)	42 (32.3)	10 (7.7)
**Agreeableness**					
	Sympathize with others’ feelings	5 (3.8)	8 (6.2)	81 (62.3)	36 (27.7)
	Do not trust others	20 (15.4)	61 (46.9)	39 (30.0)	10 (7.7)
**Conscientiousness**					
	Thorough	3 (2.3)	8 (6.2)	70 (53.8)	49 (37.7)
	Careless	21 (16.2)	60 (46.2)	39 (29.9)	10 (7.7)
**Neuroticism**					
	Relaxed most of the time	8 (6.1)	27 (20.8)	72 (55.4)	23 (17.7)
	Get nervous easily	9 (6.9)	27 (20.8)	66 (50.8)	28 (21.5)
**Openness to new experiences**					
	Have a vivid imagination	6 (4.6)	31 (23.8)	60 (46.2)	33 (25.4)
	Conservative	8 (6.2)	29 (22.3)	71 (54.6)	22 (16.9)

Table 3 shows the results of parameter estimates for time-independent covariates. Participants’ gender, age, and BMI had no significant association with the odds of reporting a mood status during the study period. Those who were relaxed most of the time seemed to have lower odds of reporting a mood status at a level k or below (OR 0.78, 95% CI 0.58-1.04, *P*=.09), indicating a marginal association between better mood and emotional stability. Being quiet (ie, "do not talk a lot"), being sympathetic to others’ feelings, and not trusting others had marginally significant associations with higher odds of reporting a mood status at a level k or below, indicating the likelihood of reporting a negative mood up to 50%.

**Table 3:** Parameter estimates for the time-independent covariates in the multilevel proportional-odds model.

Covariate of interest			Estimate (SE)	OR^a^ (95% CI)	*P*
Gender (female as reference)	Male		-0.114 (0.134)	1.12 (0.86-1.46)	.40
**Age group (26≤age≤59 years as reference) in years**					
	<26		0.084 (0.121)	0.92 (0.73-1.17)	.49
	>59		0.233 (0.242)	0.79 (0.49-1.27)	.34
**BMI** ^b^ **(18.5≤BMI<24 kg/m^2^ as reference), kg/m^2^**					
	<18.5		0.089 (0.159)	0.91 (0.67-1.25)	.58
	24≤BMI<27		-0.013 (0.160)	1.01 (0.74-1.38)	.94
	≥27		-0.105 (0.234)	1.11 (0.70-1.76)	.65
**Big Five personality items**					
	**Extraversion**				
		Outgoing and sociable	-0.109 (0.137)	1.12 (0.85-1.46)	.43
		Do not talk a lot	-0.214 (0.123)	1.24 (0.97-1.58)	.08
	**Agreeableness**				
		Sympathize with others’ feelings	-0.409 (0.218)	1.50 (0.98-2.31)	.06
		Do not trust others	-0.215 (0.123)	1.24 (0.97-1.58)	.08
	**Conscientiousness**				
		Thorough	0.312 (0.225)	0.73 (0.47-1.14)	.17
		Careless	-0.093 (0.119)	1.10 (0.87-1.39)	.43
	**Neuroticism**				
		Relaxed most of the time	0.254 (0.150)	0.78 (0.58-1.04)	.09
		Get nervous easily	0.016 (0.140)	0.98 (0.75-1.30)	.91
	**Openness to new experiences**				
		Have a vivid imagination	0.092 (0.134)	0.91 (0.70-1.19)	.49
		Conservative	-0.042 (0.129)	1.04 (0.81-1.34)	.74

^a^Odds ratio (OR).

^b^Body mass index (BMI).

Compared to time-independent covariates, several time-dependent covariates were more closely associated with the odds of reporting a mood status at a level k or below (see Table 4). The odds ratio estimate of 0.36 (*P*<.001) for those who slept very well, for example, indicates that they had a 64% reduction in odds compared to those who slept just fairly, after taking the length of sleep, diet, and other lifestyle factors into account. In contrast, participants who slept very poorly reported odds of moods being at or below a level that was 147% greater than the odds of those who slept fairly (*P*<.001). This finding supports our expectation of a strong relationship between the quality of sleep and one’s mood in daily life. Participants’ moods were also closely associated with longer duration of physical exercise. The odds of reporting mood status at a level k or below was 0.86 (95% CI 0.76-0.98, *P*=.021) for people who exercised more than 60 minutes a day, compared to those who did not exercise at all. To a lesser degree, exercising for 1 to 30 minutes or 31 to 60 minutes per day also helped—OR 0.93 (95% CI 0.86-1.00) and 0.91 (95% CI 0.83-1.00), respectively.

**Table 4:** Parameter estimates for the time-dependent covariates in the multilevel proportional-odds model.

Covariate of interest			Estimate (SE)	OR^a^ (95% CI)	*P*
**Sleeping quality (Fair as reference)**					
	Very poor		-0.903 (0.121)	2.47 (1.94-3.13)	<.001
	Poor		-0.364 (0.052)	1.44 (1.30-1.59)	<.001
	Good		0.529 (0.040)	0.59 (0.54-0.64)	<.001
	Very good		1.036 (0.077)	0.36 (0.31-0.41)	<.001
**Length of sleep (6-8 hours as reference), hours**					
	<6		0.489 (0.305)	0.61 (0.34-1.12)	.11
	>8		-0.110 (0.153)	1.12 (0.83-1.51)	.47
**Duration of physical exercise (None as reference), minutes**					
	1-30		0.075 (0.041)	0.93 (0.86-1.00)	.07
	31-60		0.093 (0.049)	0.91 (0.83-1.00)	.06
	>60		0.149 (0.064)	0.86 (0.76-0.98)	.02
**Diet (None as reference within each category), servings**					
	**Meat**				
		0-2	0.032 (0.049)	0.97 (0.88-1.07)	.51
		≥2	0.065 (0.057)	0.94 (0.84-1.05)	.25
	**Seafood**				
		0-2	0.047 (0.036)	0.95 (0.89-1.02)	.19
		≥2	0.061 (0.067)	0.94 (0.83-1.07)	.36
	**Milk and cheese**				
		0-2	-0.029 (0.035)	1.03 (0.96-1.10)	.41
		≥2	0.037 (0.066)	0.96 (0.85-1.10)	.57
	**Whole grains and rootstock**				
		0-2	0.057 (0.036)	0.94 (0.88-1.01)	.12
		≥2	-0.023 (0.063)	1.02 (0.90-1.16)	.72
	**Fried food**				
		0-1	0.000 (0.050)	1.00 (0.91-1.10)	.997
		≥1	0.081 (0.040)	0.92 (0.85-1.00)	.04
	**Sugary drinks**				
		0-2	0.013 (0.036)	0.99 (0.92-1.06)	.72
		≥2	0.133 (0.063)	0.88 (0.77-0.99)	.04
	**Dessert**				
		0-1	0.081 (0.047)	0.92 (0.84-1.01)	.08
		≥1	0.025 (0.038)	0.98 (0.90-1.05)	.51
	**Vegetables and fruits**				
		0-4	0.175 (0.085)	0.84 (0.71-0.99)	.04
		≥4	0.240 (0.093)	0.79 (0.66-0.94)	.01
**Day of the week (Tuesday-Friday as reference)**					
	Monday		-0.010 (0.040)	1.01 (0.93-1.09)	.79
	Saturday, Sunday		0.177 (0.034)	0.84 (0.78-0.90)	<.001
ILI^b^ symptoms (Yes as reference)	Without ILI		0.109 (0.048)	0.90 (0.82-0.98)	.02
**Distance away from residence (<1 km as reference), km**					
	1-9		0.036 (0.051)	0.96 (0.87-1.07)	.48
	10-49		0.150 (0.055)	0.86 (0.77-0.96)	.006
	≥50		0.336 (0.074)	0.71 (0.62-0.83)	<.001
**Number of people contacted (<5 as reference), n**					
	5-9		-0.072 (0.066)	1.07 (0.94-1.22)	.27
	≥10		-0.048 (0.068)	1.05 (0.92-1.20)	.48

^a^Odds ratio (OR).

^b^Influenza-like illness (ILI).

**Figure 2:**
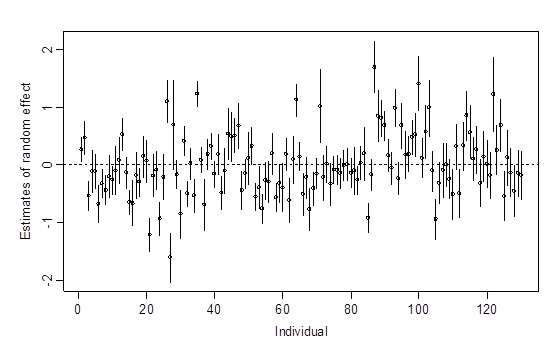
Estimates of random effects with 95% confidence intervals for the random components of the 130 individuals representing the participants' threshold deviations in the multilevel proportional-odds mixed model.

Some types of diets were positively related to the mood, too. Participants who ate four servings or more of vegetables or fruits had lower odds of reporting a mood status at a level of k or below (OR 0.79, 95% CI 0.66-0.94, *P*=.01) than those who did not eat any vegetables or fruits. Eating fewer servings of vegetables or fruits also resulted in lower odds (OR 0.84, 95% CI 0.71-0.99, *P*=.04). Those who had two bottles or more of sugary drinks had lower odds compared with those who did not have such drinks (OR 0.88, 95% CI 0.77-0.99, *P*=.04). Moreover, eating less than one serving of dessert was also marginally associated with daily mood (OR 0.92, 95% CI 0.84-1.01, *P*=.08). One surprising finding was that taking at least one serving of fried food was also linked to a somewhat better mood (OR 0.92, 95% CI 0.85-1.00, *P*=.04). Participants tended to have lower odds of reporting their mood status at a level of k or below on weekends (OR 0.84, 95% CI 0.78-0.90, *P*<.001). Traveling away from home by at least 50 kilometers was associated with significantly lower odds (OR 0.71, 95% CI 0.62-0.83, *P*<.001), as was traveling 10 to 49 kilometers away (OR 0.86, 95% CI 0.77-0.96, *P*=.006). As expected, people who did not suffer from any symptoms of influenza-like illness (ILI) tended to experience a better mood (OR 0.90, 95% CI 0.82-0.98, *P*=.02).The variance estimate, σ^2^_a_=0.327, for the random components representing variation of these thresholds among the n individuals, was not negligible. As shown in Figure 2, we saw a lot of random-effect estimates of this variance component deviated away from the zero mean. This indicates that there is still some uncertainty that remains unexplained by the covariates considered in the model. The estimate of variance, σ^2^_b_, for the other four variance components was 1.587. This result was expected because participants’ reports on mood status were affected by their previous reports to various degrees, as shown in Table 5.

**Table 5:** Number of mood changes reported in two consecutive diary entries by the 130 participants during the study period of 184 days.

Response for current report, n (%)	Response for previous report, n (%)			
	Poor	Fair	Good	Very good
Poor	142 (40.3)	138 (3.99)	57 (1.45)	11 (1.02)
Fair	153 (43.5)	2834 (81.91)	442 (11.24)	36 (3.32)
Good	43 (12.2)	458 (13.23)	3262 (82.96)	164 (15.14)
Very good	14 (4.0)	30 (0.87)	171 (4.35)	872 (80.52)
Total	352 (100)	3460 (100)	3932 (100)	1080 (100)

Among the participants who reported having a poor mood, about 40% reported the same level during the following day, 43% moved to fair, and 12% moved to good. On the other end, among those who reported a very good mood, about 81% felt the same in the following report, 15% changed to good, and 3% changed to fair. For participants who reported fair or good moods, about 82% to 83% retained the same feeling the following day, while about 11% to 13% tended to swing between these two levels. This phenomenon had been adjusted by the four random components with effects estimates b_1_ = -1.43, b_2_ = -0.8, b_3_ = 0.33, and b_4_ = 1.88 in the proportional-odds model.

## Discussion

### Principal Findings

This study provides clear evidence that an individual’s mood can be associated with health behaviors and activities in everyday life. Our findings are based on longitudinal diaries collected through the user-friendly, online ClickDiary platform, which allows participants to select their mood status and health behaviors by simply clicking appropriate response items in the respective diaries. Using this platform, participants can record their daily activities during the past 24 hours at their convenience. The design should have substantially reduced recall bias.

On average, it takes only about one minute to complete one health diary entry. Such a low-demanding task helps keep users participating in the study for a longer period of time. In addition, the visualization of their own diary entries and the random drawings help new participants sign up and existing participants to remain committed. Long-term data on variations in participants’ moods and health behaviors are essential for understanding the dynamic interactions between the two. In contrast, a cross-sectional or short-term follow-up study design would not allow researchers to collect the wealth of information on the daily variations provided by each participant. The advantages of such longitudinal data can be further examined by comparing our findings to those of previous studies that focus on emotions and health behaviors.

### Comparison With Previous Research

Earlier studies showed that a higher BMI or being overweight was closely linked to negative affect among young adults and adverse psychosocial outcomes among grade-school children [7,28]. Among adolescents with excess weight, BMI was also a good predictor of emotion-driven impulsivity and cognitive inflexibility [29]. Our results, however, do not show that BMI has a significantly negative correlation with an individual's mood. Note that BMIs for most of the individuals in this study were within the normal range. This may be a reason for not finding a significant association in our analysis.

Most of our participants perceived themselves as being agreeable and conscientious. From the parameter estimates in the model, we found that agreeableness tends to be negatively correlated with a person’s mood, while relaxation is positively correlated. The finding differs from that of a study conducted in Finland [8], which showed that agreeableness is associated with higher positive and lower negative effects on mood, and that neuroticism predicts higher negative and lower positive effects on mood. The exact underlying reasons for the differences need to be further examined. The Finnish study, however, employed a very different data collection approach and sample groups—it recruited 106 university students aged 19 to 35 and used portable devices, such as mobile phones, to collect information 10 times per day for 1 week. By contrast, the ClickDiary participants came from different backgrounds, including students, full-time workers, housewives, and retired persons, between the ages of 20 and 67. Our measure of daily mood was recorded once a day for an average of 69 days. While the Finnish study collected the mood changes detailed within each day, our study collected the mood variations on different days over a longer period of time.

With regard to daily diets, we found that eating vegetables and fruits is related to better mood, which is consistent with findings from previous studies [6,7,30]. The possible biological mechanism is from the polyphenols found in fruits and vegetables. They can battle oxidative stress and help stimulate the activation of the neural molecules that aid in synaptic plasticity, which is important for cognitive function [31]. Currently, there are over 8000 polyphenolic compounds that have been identified in the world. For example, cocoa polyphenols have been shown to cause positive mood in one randomized study [32]. We also found, however, that eating certain unhealthful foods, such as fried food and sweetened beverages, is correlated to a slightly positive mood. Because it is difficult to differentiate the temporal order between eating behaviors and mood changes, we cannot conclude that such unhealthful foods actually trigger more positive emotions. Although such foods might play a role in promoting a good mood, we need further experimentation and validation.

Our study confirms that the participants are clearly in a better mood on the weekends, as well as when they travel farther away from their homes. Those without ILI symptoms are also happier than their counterparts with ILI symptoms. Those who exercise more also tend to be in a better mood compared to those who do not exercise, a finding consistent with those of earlier studies [18,33,34]. In addition, the longer one exercises, the better his or her mood becomes.

Most important, we found the quality of sleep to be a strong factor in distinguishing how one’s mood changes from day to day. Having slept well or not during the previous night has a clear effect on a person’s mood the following day. As also found in other studies [19,35], better-quality sleep clearly leads to a more positive mood the following day.

### Limitations

Despite various interesting findings from this study, some limitations remain to be addressed. First, our sample of participants was not representative of Taiwan’s population. Due to the longitudinal nature of diary studies, we required long-term and demanding commitment from our participants. After further screening for complex data analyses, only 130 participants met our strict threshold. Even though we tried to recruit participants through various channels, the demographic distribution of our participants was still skewed to females and young adults. Such a skewed sample tends to be common in many online surveys as well [7,36,37], which might be due to health issues and patterns of computer use. As a result, we cannot infer our findings to the general population, but the internal validity of the study is retained.

Second, we cannot make definite causal inferences between moods and health behaviors. Our ClickDiary platform requires participants to record their moods and health behaviors during the past 24 hours, but the temporal order of emotions and health behaviors remains unclear. Therefore, we can identify only the overall mood of the participant on a given day and his or her corresponding health behaviors on the same day. In addition, there are still many uncollected factors such as working stress and other life behaviors affecting people’s moods within the day. In the current study design, we were not able to capture and adjust all these factors in the model.

Third, some participants’ contact and health diaries may not have been complete. In this study, the average length of participation was about 69 days during the 184 days of follow-up. Unlike previous diary research that managed to collect complete information about one’s daily contacts during the full periods of study [38,39], the diary records of some of our participants may have lagged at different intervals. The resulting sporadic records may have somewhat inhibited further analyses that required continuous time-series data. The study is still ongoing, however, and mobile apps for the iOS and Android systems have been released in January 2015. With participants from more diverse sources, a longer observation period, and more complete diary entries on a continuous basis, we expect to reexamine and further validate our current findings in the near future.

### Conclusions

Sleeping well, eating more fruits and vegetables, and exercising longer all contribute significantly to improving a person’s mood in everyday life. Using our online ClickDiary program, which helps reduce the recall bias associated with traditional one-shot surveys, we collected data on a daily basis to carefully identify the links between health behaviors and mood.

## Abbreviations

BMI: body mass index
ILI: influenza-like illness
IPIP: International Personality Item Pool
IRB: Institutional Review Board
OCEAN: openness, conscientiousness, extraversion, agreeableness, neuroticism
OR: odds ratio
ROC: Republic of China
